# Development and multi-center validation of a software program, Ofeye 3.0, for automated all-inclusive vertebral fracture detection with chest/abdominal CT images

**DOI:** 10.1016/j.jot.2025.08.011

**Published:** 2025-09-13

**Authors:** Ben-Heng Xiao, Zhen-Hua Gao, Cai-Ying Li, Xiao-Ming Leng, Er-Zhu Du, Jian-Bing Ma, Fu-Shan Liu, Jing-Shan Gong, Zhi-Guo Ju, Ming-Yuan Yuan, Hui-Ming Zhu, Michael S.Y. Zhu, Timothy YC. Kwok, Yì Xiáng J. Wáng

**Affiliations:** a Department of Imaging and Interventional Radiology, Faculty of Medicine, the Chinese University of Hong Kong, Hong Kong Special Administrative Region of China; b Department of Radiology, The First Affiliated Hospital, Sun Yat-Sen University, Guangzhou, China; c Universal Medical Imaging Diagnostic Center of Guangzhou, Guangzhou, China; d Department of Radiology, Dongguan Traditional Chinese Medicine Hospital, Dongguan, Guangdong province, China; e Department of Radiology, The First Hospital of Jiaxing, the Affiliated Hospital of Jiaxing University, Jiaxing, Zhejiang, China; f Shandong Healthcare Group Xinwen Center Hospital, Tai'an, Shandong, China; g Department of Radiology, Shenzhen People's Hospital, the Second Clinical Medical College, Jinan University, Shenzhen, China; h College of Medical Imaging, Shanghai University of Medicine and Health Science, Shanghai, China; i Department of Radiology, Affiliated Zhoupu Hospital, Shanghai University of Medicine and Health Sciences, Shanghai, China; j The Sixth People's Hospital of Huizhou, Huizhou, Guangdong, China; k Yingran Medicals Co. Ltd, Hong Kong Special Administrative Region of China; l JC Centre for Osteoporosis Care and Control, Faculty of Medicine, the Chinese University of Hong Kong, Hong Kong Special Administrative Region of China

**Keywords:** Osteoporosis, Vertebral fracture, Machine learning, Tomograph

## Abstract

**Background:**

Missing report for fragility vertebral fracture (VF) on chest/abdominal CT is common when the indication is not spine disorders. This represents a missed opportunity to alert patients to take preventive measures to improve bone health and prevent further severe fractures. In this study, we aim to develop a software program for automated detection of VF with existing chest/abdominal CT scans and validate its detection performance.

**Methods:**

An automated sagittal Central Slab reconstruction (CSR) method for CT axial images was developed. For reference VF reading*,* VFs inclusive of those of with <20 % vertebral height loss and those of endplate fracture with minimal vertebral height loss were identified. VFs were also differentiated from osteoarthritic wedging and endplatitis short vertebrae. Prior knowledge of VF detection models for lateral radiograph were transferred to a new ‘Ofeye 3.0’ model optimized for VF detection on CT image. Training CT images were obtained from nine centers, totaling 3313 cases without VF and 835 cases with VF. For external validation, CT images were from five centers totaling 732 cases without VF and 224 cases with VF.

**Results:**

The automated CSR method showed advantages in demonstrating structural changes of the endplate and adjacent structures. For detecting VF in chest/abdominal CT scans, counting case-by-case and compared with the reference reading, the average performance of Ofeye 3.0 was accuracy 0.967, sensitivity 0.906, and specificity 0.986. Most of false negative or false positive cases were minimal or mild VF, or with image artifacts, or with VF close to the peripheral of CSR images.

**Conclusions:**

Despite the challenging requirements for the software to detect all-inclusive VF, our results compare favorably with other published automated VF detection models.

**The translational potential of this article:**

We developed a software program for automated all-inclusive VF detection on chest and/or abdominal CT image data and conducted a multi-center external validation study. This software is proved to have high VF detection precision. By alerting patients of the VFs likely related to osteoporosis and in turn the patients taking measures to prevent further fracture, the integration of this software into radiological practice will improve patient outcomes and reduce healthcare costs.

## Graphical abstract

**Figure:**
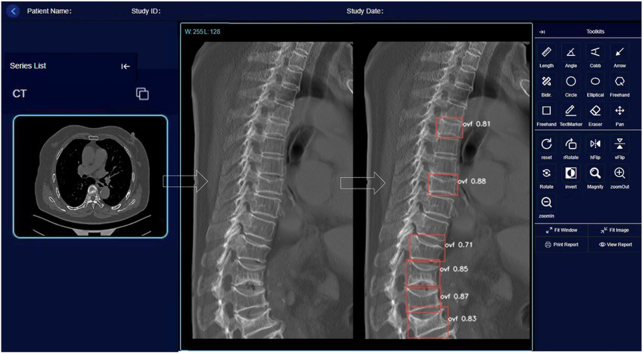

## 1 Introduction

Assessment of vertebral fracture (VF) status, in addition to BMD (bone mineral density), aids in predicting fracture risk in older populations. Siris et al. [1] reported that at any given BMD T-score, the risk of incident vertebral, non-vertebral, and any fracture among postmenopausal women depended heavily on prevalent radiographic vertebral fragile fracture (VFF) status. Johansson et al. [2] reported that, in older women and after adjustment for clinical risk factors and BMD, grade-1 VFFs identified on lateral spine imaging with dual-energy x-ray absorptiometry are associated with incident major osteoporotic fractures. It is well documented that incident VFFs shown on chest and abdominal CT examined are associated with increased further hip fragile fracture (HFF) risk. Buckens et al. [3] studied chest CT examinees of ≥40 years old and those later hospitalized for HFF during a median follow‐up of 4.4 years. Genant grading was used to evaluate VFF [4]. After adjustment for age and gender, the presence of any VFF was associated with future hip fracture by a HRadj (adjusted Hazard Ratio) of 3.1. For men, the HRadj was 2.8, whereas it was 3.5 for women. Having a mild VFF grade was associated with a 2.4‐fold increased future fracture risk, a moderate grade VFF with a 4.8‐fold increased risk, and a severe grade VFF with a 6.7‐fold increased risk. For cumulative fracture grade, compared with a score of 0, a score of 1–3 conferred a HRadj of 2.7, a score of 4–6 conferred a HRadj of 4.8, and a score of ≥7 conferred a HRadj of 11.2. Lee et al. [5] studied 204 HFF patients and 204 controls without fracture patients. HFF patients had abdominal CT performed within 6 years of the date that HFF occurred, 204 control subjects were from a screening CT colonography database. The mean interval from CT to HFF was 25.3 ± 20.8 months. The mean age of the case patients and control subjects was 74.3 ± 11.8 and 72.9 ± 10.65 years, respectively. In the cohort with future HFF, at least one prevalent moderate or severe VF was seen in 37 case patients who had a previous CT examination performed (18.1 %), compared with five of 204 control subjects (2.5 %). Skjødt et al. [6] identified CT scans including the chest and/or abdomen of 2000 consecutive men and women aged 50 years or older, and subjects were followed for up to 7 years. The risk of subsequent HFF had HRadj of 3.02. All these data show the role of VFF as early manifestation of the deteriorated bone microarchitecture. In addition to high mortality (the one-year mortality after HFF of around 20 %), HFF patients are associated with poor functional recovery and often are unable to resume their pre-fracture function with a consequent deterioration in quality of life [[7], [8], [9]].

When the indication is not spine disorders, previous studies have shown that the majority of VFFs shown on chest/abdominal CT are not reported [[10], [11], [12], [13]]. For example, Mitchell et al. [12] described a UK study where 732 patients over the age of 50 with HFF were identified. 157 patients had previously undergone a radiological procedure involving the spine, and VFFs were identified in 65/157 (41 %). 54 % of VFF fractures were not reported by the radiologist. In images where the most severe grade of VF was grade 1, only 7 % of VFs were reported. Reporting VFFs is an important component of the International Osteoporosis Foundation (IOF) Best Practice Framework for secondary fracture prevention [14]. Knowing that a patient has a VFF will also give more accurate assessments of future fracture risk using the WHO Fracture Risk Assessment Tool. Reporting of serendipitous VFFs by radiologists, and appropriate follow-up by the referring clinician, can help to reduce the number of hip fractures. Because the missing report for VFF on chest/abdominal CT is widespread, computer aided automated detection of VF is highly useful. In this study, we developed a software program for automated detection of VF with existing chest/abdominal CT scans and validated its detection performance using multi-center external CT data.

## 2 Methods and materials

### 2.1 Sagittal central slab reconstruction (CSR) of axial CT images

**Fig. 1:**
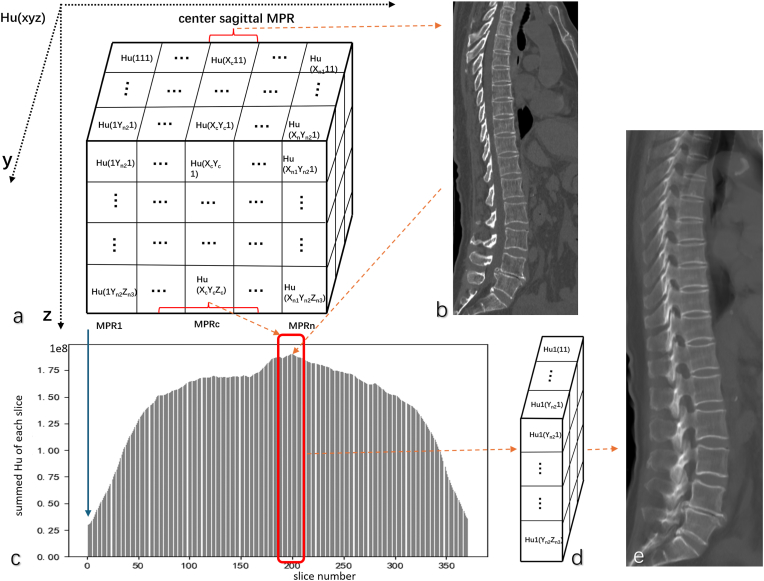
Illustration of CSR (central slab reconstruction) method. A: the cube is a 3D data matrix of chest or abdominal CT images, which can be obtained by superimposing the cross-sectional images in the z-axis direction; Hu(xyz) represents the Hu value (Hounsfield unit) in the (x, y , z) coordinates in the 3D matrix. Conventional MPR (multi-planar reconstruction) is used to reconstruct a whole series of single voxel based sagittal images (B). The histogram method is used to analyse all sagittal MPR images along the x-axis (C). The X-axis in c is the MPR image ‘index number’, and the Y-axis in c is the sum of the Hu values of the MPR image. Bone tissue has a higher Hu value in CT images than other tissues, thus the closer to the middle of the spine the sagittal MPR image, the higher the Hu value of the MPR image. The MPR image with the highest Hu is the image index of the middle spine. Adjacent 20 slices to the left of the central MPR image and adjacent 20 slices to the right of the central MPR image (total n = 40) are selected and projected them along the x-axis, so that most of the middle spinal tissue were included in it (D). The projection calculation method is as shown in formula 1, $HuC\left(ij\right)=\sum_{k=slice\;number\;of\;MPRc-20}^{slice\;number\;of\;MPRc+20}Hu\left(kij\right)$ (1) Where i and j are the coordinates of the CSR image, and k is the slice number of MPR image. Thus, the Hu value matrix of the reconstructed target image was obtained, and then the Hu value matrix was converted to a natural image range of 0–255 to obtain the aggregated reconstructed sagittal image, and the final images were in the PNG (Portable Network Graphics) format (E).

**Fig. 2:**
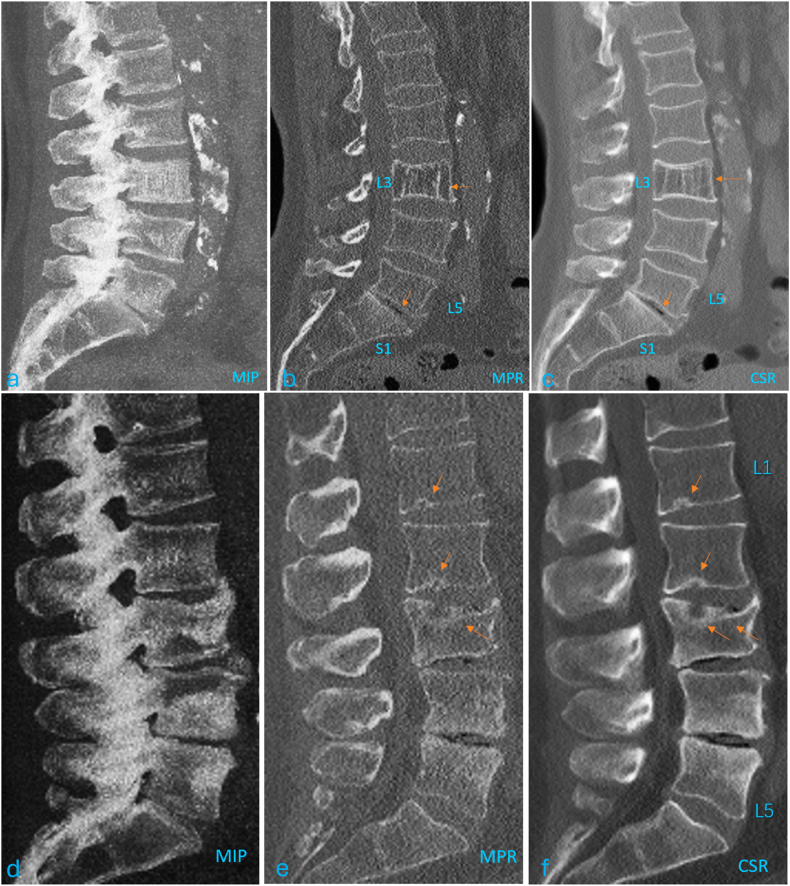
A comparison of MIP (maximum intensity projection, a, d), MPR (multi-planar reconstruction, b, e), and CSR (*Central Slab reconstruction,* c, f) in demonstrating middle spine structures. A1, B1, C1 show L3 vertebral osteoporotic change (arrow), L5/S1 disc space minimal amount of air collection (arrow), and S1 higher density are best shown with CSR image. b, c, e, f show L1, L2, L3 Schmorl's nodes (arrow), L3 upper endplate depression, L4 and L5 Modic change, and L4/L5 and L5/S1 disc space minimal amount of air collections are best shown with CSR image.

Reconstructing sagittal image using the CSR method is explained in Fig. 1 [15]. For CT image pre-processing, the first step was to remove personal information contained in DICOM format images; the second step was to adjust the window width and window level of the CT image, with the window width set to 1500 and the window level set to 300. Compared with commonly used maximum intensity projection (MIP) and multi-planar reconstruction (MPR), CSR CT image demonstrates more vertebral structural details as shown in Fig. 2.

### 2.2 VF assessment

The morphology of thoracic and lumbar vertebrae was evaluated for VFs inclusive of those of <20 % vertebral height loss and those of vertebral endplate/cortex fracture (ECF) with minimal vertebral height loss, according to the ESSR (European Society of Musculoskeletal radiology) recommendation [16,17]. It was considered that the extent of vertebral height loss is not an absolute criterion to differentiate VFF and other VFs [16,17]. Nonfractural changes/deformities of the vertebrae shape were differentiated from VF [[18], [19], [20], [21]]. Osteoarthritis (OA) wedging (OAw) was diagnosed according to Abdel-Hamid Osman et al. [20], typically appearing as anterior wedging and intervertebral disc space narrowing and involving at least two adjacent vertebrae. OA wedging is also without apparent endplate depression and is not associated with an increased further VF risk. Acquired short vertebrae (SV) are vertebral deformities with middle vertebra height and anterior vertebra height reduced to a similar extent, and these vertebrae commonly have an anteroposteriorly elongated shape [21]. SV is not associated with an increased further VF risk. It is also noted that while congenital Schmorl nodes are not intrinsically associated with low bone strength, acquired Schmorl nodes are associated with endplate fracture ECF and is a sign of VF [22]. For all vertebrae with VF, the fractured vertebrae on the CSR sagittal image were all manually labeled to obtain the position coordinates and size in the image. All images were triple-read by three trained readers (BHX, CYL, and YXJW, with one being a senior radiologist), with consensus all reached. It has been subjectively noted that CSR CT image is advantageous in demonstrating structural changes of the endplate and adjacent structures, including endplatitis [21].

### 2.3 Model training and optimization

To develop VF detection model, we used both radiograph and sagittal CSR CT images. Radiographs were used to develop the ‘base model’ and included lateral chest radiographs and lateral spine radiographs from 16 centers, containing 5970 cases in total with 1404 cases having VF. The details of lateral chest radiograph and some spine radiograph data and the processing steps have been published earlier [23]. The model for thoracic spine radiograph was termed Ofeye 1.0. Based on Ofeye 1.0 and followed the same principles, additional 638 lateral lumbar radiograph cases (540 cases with VF, with selection targeted toward traumatic VF with minimal or mild deformity) from three centers were trained for a ‘Ofeye 2.0’ model to read lateral lumbar radiograph (including traumatic VF). Prior knowledge of Ofeye 1.0 model and Ofeye 2.0 model were then transferred to a new ‘Ofeye 3.0’ model optimized for VF detection of CSR CT image of the spine. Training CT images (including internal validation CT) were from nine centers with scans covering chest, abdomen or spine (Table 1). CT data selection was on a random basis. The slice thickness was required to be ≤ 3 mm, and the study subjects were required to be ≥ 50 years. The scanning protocol parameters of the CT data are shown in Table 2, we also provide more detailed information in the supplementary material.

**Table 1:** **CT data used for Ofeye 3.0 model training**. The CT images were of mixture of Siemens, GE, Philips, and Toshiba format.

Sources	CT scan region	age	Total cases	VF cases
Center 1	Chest	≥65 years	200	126
Center 2	Abdomen	≥65 years	416	253
Center 3	Chest	≥65 years	100	72
Center 4	Chest or Abdomen	≥65 years	200	130
Center 5	Abdomen	≥50 years	825	89
Center 6	Spine	Mixed age	300	85
Center 7	Abdomen	Mixed age	201	26
Center 8	Chest	Mixed age	771	5
Center 9	Spine	Mixed age	125	46
Total			4148	832

**Table 2:** **Scanning procotool** (*UIH, United Imaging Healthcare; SIEMENS, Siemens Healthineers; TOSHIBA, Toshiba Medical Systems Corporation; GE, GE MEDICAL SYSTEMS; CANON, Canon Medical Systems).

	Manufacture	X-Ray Tube Voltage (Kv)	x-ray tube current (mA)	Thickness (mm)	Pitch	Pixel spacing (mm)	Scanner types
Center 1	SIEMENS/TOSHIBA/UIH	80–140	91–1566	0.62–5.0	0.8–3 0.0	0.51–0.82	chest/abdomen
Center 2	SIEMENS/GE	100–120	59–453	0.62–1.25	0.8–1.0	0.57–0.96	chest/abdomen
Center 3	SIEMENS/GE/TOSHIBA/PHILIPS/CANON	70–140	50–1997	0.50–5.0	0.27–1.90	0.40–1.95	chest/abdomen/heart
Center 4	SIEMENS	100–130	121–1891	0.60–3.0	0.23–1.20	0.30–0.89	chest/abdomen/spine/heart
Center 5	SIEMENS/GE	120–140	350–664	1.0–1.25	0.55–1.37	0.57–0.98	Chest/pelvis/abdomen

The CSR CT images were divided into two parts: a training set and an internal validation set. The training data set contained 2520 images with VF, and the internal validation dataset contained 256 images with VF. To mitigate the problem of model toward non-VF detection, when in training stage, we used the 2520 images which contain VF, to further optimize the model CT images of 92 cases of healthy volunteers were added to the validation data to balance the positive and negative samples.

Before inputting into the model, the CSR images were standardized to 512 × 512 in size, with those larger than 512 × 512 cropped and those smaller than 512 × 512 padded with zeros at the image border. We used a single-stage target detection algorithm, the VF target detection problem was regarded as a regression problem. The category and bounding box of the target were predicted directly from the image. The input image was divided into grids of different sizes, and each grid predicted a fixed number of bounding boxes and category probabilities. In the post-processing process of target detection, multiple target boxes were screened, the non-maximum suppression algorithm was used to optimize VF detection.

**Fig. 3:**
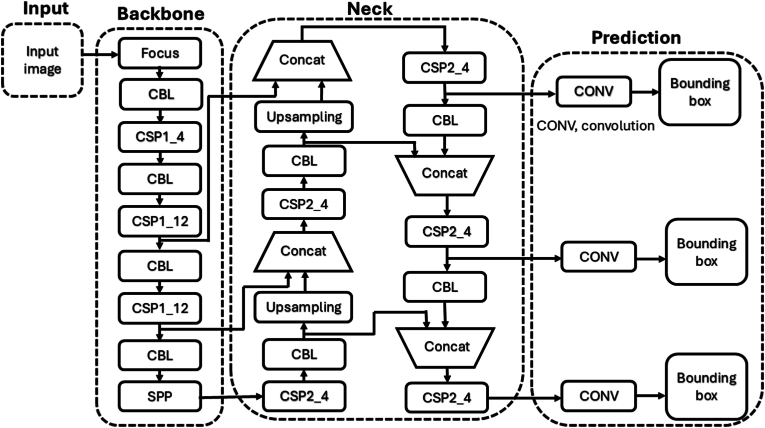
Structure of VF detection model.

**Fig. 4:**
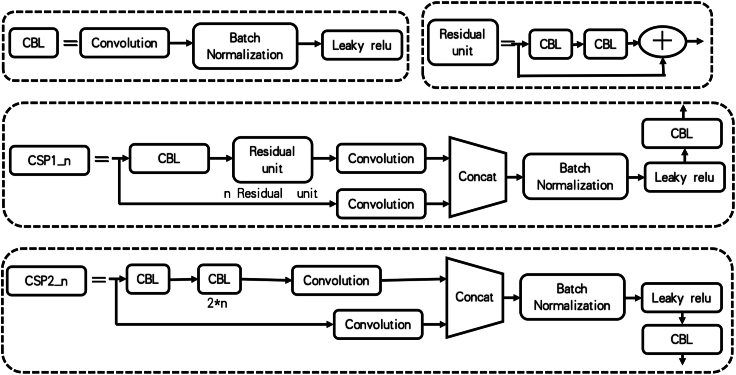
Components structure of CBL, Residual unit, CSP1_n and CSP2_n.

**Fig. 5:**
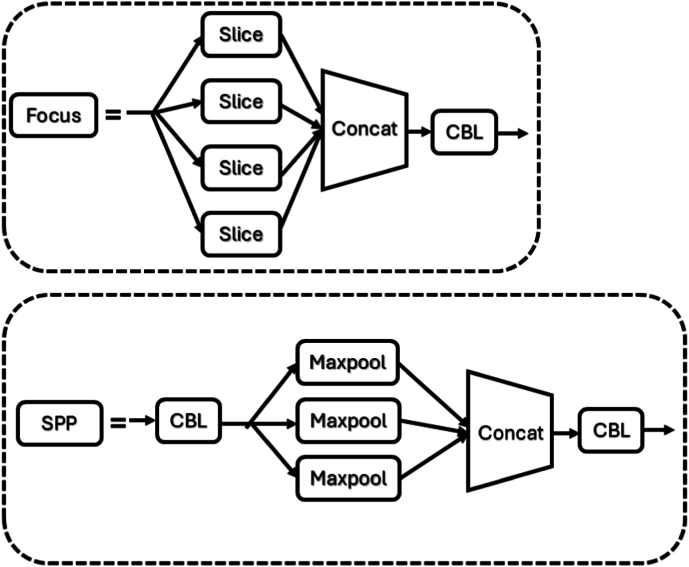
Components structure of Focus and SPP.

We used the yolov5x framework to train our VF detection model, the algorithm structure was composed of three parts (details as shown in Fig. 3), backbone, neck, and prediction [24], backbone and neck parts were responsible for feature extraction, feature fusion, and prediction part responsible for predicting detection bounding box, each part was made up of different components (details as shown in Fig. 4, Fig. 5). Backbone includes Focus, CBL, CSP1_4, CSP1_12 and SPP modules, CBL ws composed of convolution layers that include convolution kernels of different sizes, batch normalization layer and leaky relu activation function, CPS1_4 include1 CBL module, 4 residual modules [25], feature merging layers, SPP include 1 CBL module, max pooling layers etc. Neck includes CBL module, CSP2_4, up-sampling layers and SPP feature merging modules. Prediction included convolution layers, totaling 444 layers.

The main training parameters were: batch size = 32, a larger batch size provides a more stable gradient estimate due to the average gradient of more samples is considered each time the weights are updated, making the training process smoother, which may lead to slightly worse model generalization ability. A smaller batch size may cause fluctuations in the training process, but sometimes it helps the model escape from the local minimum and find a better global minimum, which may improve generalization ability. However, the training is unstable and may require more epochs to converge, epoch = 600, with fewer epochs, the model may not learn sufficiently, resulting in underfitting, especially on complex tasks or large data sets. With more epochs, the model may overfitting, that is, it performs well on the training set, but the performance on the validation set or test set degrades, and the initial learning rate = 0.01, a higher learning rate results in a larger weight update amplitude and faster model convergence, but it may cause unstable training or even failure to converge. A lower learning rate results in a smaller weight update amplitude and more stable training, but it converges slowly and may fall into a local minimum. As the number of model iterations increased, the cosine annealing function was used to gradually reduce the learning rate. The dropblock function was used to alleviate the problem of model overfitting. The loss function was DIOU_Loss (distance intersection over union loss), and the (Stochastic Gradient Descent) SGD gradient descent algorithm was used for parameter optimization. Model training was performed using Ubuntu 18.04.4 LTS, python (v3.6.12), pytorch (v1.7.1) machine learning libraries, Nvidia A100 (40 GB memory) Tensor Core Graphics Processing Unit, and Xenon E5-2698 v4 2.2 GHz, 20 Cores Central Processing Unit. When the model loss function could not be further decreased, the optimal training model, which we called ‘VF CT-model Ofeye 3.0’, was obtained.

### 2.4 Multi-center external validation

For external validation, in total 958 cases were provided by five external centers. Two cases were very low quality and thus excluded, finally 956 cases were utilized (Table 3). A two-step approach was adopted to test external datasets. For each case, the first step was to label a vertebra with VF when the confidence probability was high (e.g., ≥0.8). Then, if there was a VF, a further lower confidence probability (e.g., ≥0.5) was applied to identify VFs in the image as many as possible. The non-maximum suppression intersection over the union threshold was 0.6 for both steps. The parameters could be adjusted depending on whether specificity or sensitivity is emphasized. In our current model, specificity was emphasized. The reference reading for CSR CT was established as described above, and all confirmed by additional radiologists from the five participating hospitals. After the CT-model reading, the cases with false positivity and false negativity were further inspected for potential causes.

**Table 3:** **CT data used for Ofeye 3.0 model externalvalidation**. The CT images were of mixture of United Imaging (center 1), GE (center 2, center 5), Toshiba (center 3), Siemens (center-4, center-5). Junc: thoracolumbar junction. Note that CT of 85 % of the cases included thoracolumbar junction which has the highest prevalence of VF. Incl: including.

	Total cases	Above Junc	Under Junc	Incl whole spine	Incl L5
Center 1	159	136 (85.53 %)	14 (8.8 %)	9 (5.66 %)	22 (13.83 %)
Center 2	219	190 (86.75 %)	5 (2.28 %)	24 (10.95 %)	28 (12.78 %)
Center 3	215	131 (60.93 %)	75 (34.88 %)	9 (4.18 %)	23 (10.69 %)
Center 4	169	161 (95.26 %)	8 (4.73 %)	0	4 (2.36 %)
Center 5	194	150 (77.31 %)	42 (21.64 %)	2 (1.03 %)	34 (17.52 %)
Total	956	768 (80.33 %)	144 (15.06 %)	44 (4.60 %)	111 (11.61 %)

## 3 Results

**Fig. 6:**
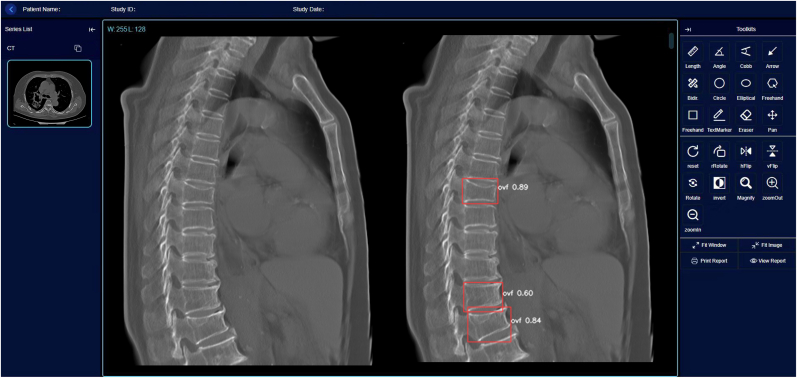
The primary Ofeye 3.0 reading output. The central window shows the sagittally reconstructed image with VF labelling (if there is/are). For each suspected VF detected, a probability is provided. The DICOM image view function of this software allows other basic functions including zoom-in, zoom-out, adjusting contrast and window levels, and distance and angle degree measurements, as shown in the tool bar on the right.

The primary Ofeye 3.0 reading output is demonstrated in Fig. 6, The central window shows the CSR and VF labelling (if VF positive). For each suspected VF detected, a probability is provided. The DICOM image view function of this software allows other basic functions including zoom-in, zoom-out, adjusting contrast and window levels, and distance and angle degree measurements, etc. Ofeye 3.0 for CT retains many functions of Ofeye 1.0 (for lateral chest radiograph). The software allows ‘batch processing’, for example, 100 CT cases can be processed in a single operation. We randomly selected 100 cases from 5 external validation datasets; average operational time from read DICOM image to output detection result was 15.4 s (test platform: Windows 11 Pro 24H2, python 3.9.0, pytorch 1.10.0 cpu edition, Intel(R) Core(TM) i5-9500 CPU @ 3.00 GHz 3.00 GHz, 16 GB RAM). This software can be integrated into hospital PACS (allowing multiple users simultaneously) or installed on a standalone workstation or personal computer. In the interaction of the Radiology Information System (RIS) or Picture Archiving and Communication Systems (PACS), the DICOM transmission protocol was used to automatically and in real time transmit CT images that meet the conditions (for example, women aged >60 years old) in the system to the image processing workstation. After model processing, the prediction results (such as whether there is a VF, the position of the VF vertebral information and the CSR reconstructed image) are returned to the RIS or PACS through hospital's local area network. Then radiologists can view the model output results in the PACS or RIS, providing them with auxiliary diagnostic information. This software can regenerate text reports with image illustrations or ‘batch report’ in an excel file.

**Fig. 7:**
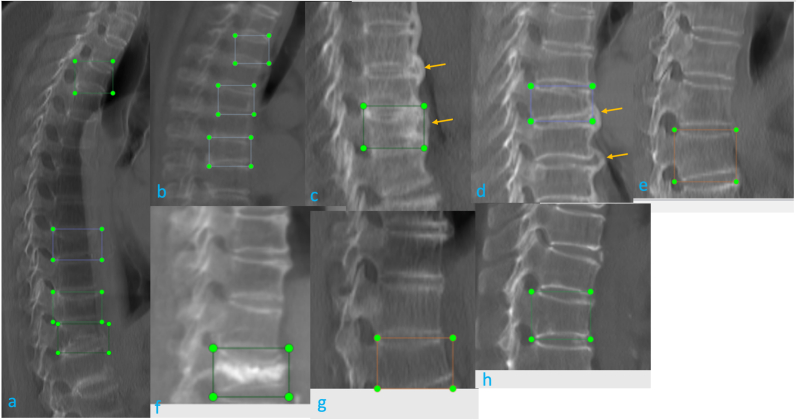
CSR CT images of 8 cases with 13 VF with false negative reading (labeled with green dotted box). A and B are associated with low signal of the vertebral, as the spine was off-center with scoliosis. C and B are associated with apparent formation of osteophytes (arrows). For E, F, G, and H, the VFs are close to the lower margin of field of view, without a full lower vertebra for reference.

**Fig. 8:**
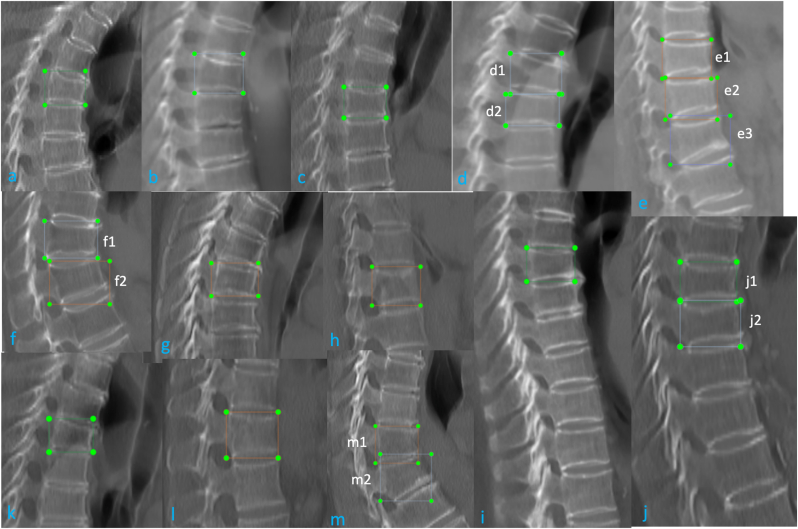
CSR CT images of 13 Cases with 19 VFs with false negative reading (labeled with green dotted box). Most of the missed VF are minimal or mild grades. In D, E, F, J, and M, missed VFs are associated with 2 or 3 adjacent VFs. Two adjacent VFs in D (d1 and d2); three adjacent VFs in E (e1, e2, and e3); two adjacent VFs in F (f1 and f2); two adjacent VFs in I (i1 and i2); and two adjacent VFs in M (m1 and m2).

**Fig. 9:**
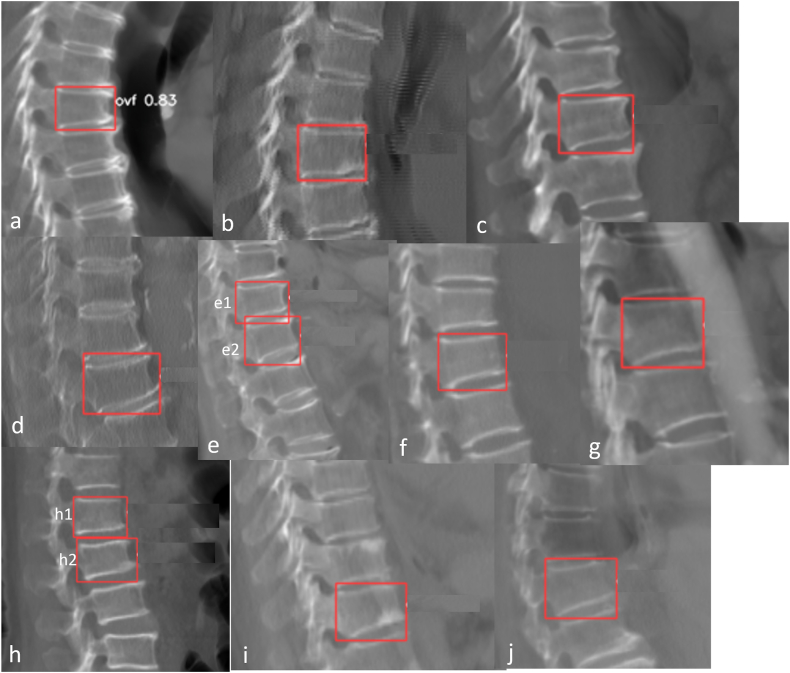
CSR CT images of false positive readings recorded in 12 vertebrae in 10 cases (A: VF probability is denoted as 0.83). Most of these vertebrae had slightly wedged shape but were not labeled as VF by the reading radiologists. The image in E is affected with two adjacent VFs (e1, e2); They could mostly be easily evaluated by a radiologist reader as being of no significance or a false positive reading.

VF detection external validation from 5 sources performance of Ofeye 3.0 is shown in Table 4. The external validation dataset from 6 manufacturers, the performance is shown in Table 5. Counting case-by-case, the average performance was accuracy 0.967, sensitivity 0.906, and specificity 0.986. Ofeye 3.0 has a false positivity rate (1.37 %). 32 OVFs in 21 cases had a false negative reading, which constituted a false negative rate of 9.37 % (21/224, counting cases) for all VF cases. Among these cases with a false negative reading, 18 VFs (56.3 %, 18/32 counting vertebrae) in 11 cases (52.4 %, 11/21 counting cases) were mild or minimal grades. For cases with ≥ moderate grades VF, 95.54 % were detected. The false negative cases and false positive cases are all shown in Fig. 7, Fig. 8, Fig. 9*.* Most of mis-diagnosed cases (false negative or false positive cases) were minimal or mild, or associated with artifacts, or close to the peripheral of CSR images. Center 1 to center 5, there are 1, 3, 3, 3, 5 cases with artifacts or peripheral, prevalence is 0.6 %, 1.36 %, 1.39 %, 1.77 %, 2.57 % respectively.CT image quality variations across centers, due to differences in pixel spacing, X-Ray Tube Voltage (Kv)/x-ray tube current (mA), slice thickness which significantly affect deep learning system outputs, low-quality images (such as high noise, large pixel spacing, large slice thickness ect.) reduce detection accuracy, vice versa.

**Table 4:** **External validation diagnostic performance of Ofeye 3.0 model counting case-by-case**. FN: false negative; FP: false positive; TN: true negative; TP: true positive; CI, confidence interval.

Data	Total	TP case	TN case	FP case	FN case	Sensitivity (95 % CI)	Specificity (95 % CI)	accuracy
Center 1	159	44	115	0	0	1 (0.899, 1)	1 (0.959, 1)	1
Center 2	219	44	170	1	4	0.916 (0.791, 0.972)	0.994 (0.962, 0.999)	0.977
Center 3	215	31	176	3	5	0.861 (0.697, 0.947)	0.983 (0.947, 0.995)	0.962
Center 4	169	27	131	2	9	0.750 (0.574, 0.872)	0.984 (0.941, 0.997)	0.934
Center 5	194	57	130	4	3	0.950 (0.851, 0.986)	0.970 (0.920, 0.990)	0.963
total	956	203	722	10	21	0.906 (0.858, 0.939)	0.986 (0.974, 0.993)	0.967

*CI, confidence interval.

95 % CI for sensitivity and specificity are calculated according to the efficient-score method described by Robert Newcombe (Newcombe, Robert G. "Two-Sided Confidence Intervals for the Single Proportion: Comparison of Seven Methods," Statistics in Medicine, 17, 857–872 (1998).), based on the procedure outlined by E. B. Wilson in 1927 (Wilson, E. B. "Probable Inference, the Law of Succession, and Statistical Inference," Journal of the American Statistical Association, 22, 209–212 (1927).).

**Table 5:** Model output performance of different manufacturers.

Data	Total	TP case	TN case	FP case	FN case	Sensitivity (95 % CI)	Specificity (95 % CI)	accuracy
SIEMENS	353	75	259	7	12	0.862 (0.767, 0.923)	0.973 (0.944, 0.988)	0.946
GE	282	60	217	1	4	0.937 (0.839, 0.979)	0.995 (0.970, 0.999)	0.982
UIH	147	42	105	0	0	1 (0.895, 1)	1 (0.956,1)	1
PHILIPS	53	8	43	1	1	0.888 (0.506, 0.994)	0.977 (0.864, 0.998)	0.962
TOSHIBA	118	18	95	1	4	0.818 (0.589, 0.940)	0.989 (0.935, 0.999)	0.958
CANON	3	0	3	0	0	None	1 (0.309, 1))	1

For the cases with only a single VF on the CSR image, the external validation results are additionally shown in Table-6. Among all VF positive cases, the proportion of single fracture is 48.2 %; and among these single VF cases, 45.4 % had only minimal grade or mild grade VF. The overall detection sensitivity for single VF cases was 87 %. Table 4 shows center-4 data was associated with less good results than the results from other centers, this was likely due to that center-4 cases were more likely to have singular VF and more likely to have minimal and mild grades VF as shown in Table-6, since the CT scan parameters of center-4 were comparable to those of other centers.

**Table 6:** **External validation diagnostic performance of Ofeye 3.0 model for cases with only single VF on CT**.^#^: No. of single VF cases and its percentage among all positive cases from each center. ¶: No. of the single VF being minimal or mild grade and its percentage among all single VF cases from each center.

	Single VF^#^	Minimal VF^¶^	Mild VF^¶^	FN case	TP case	Sensitivity
Center 1	19 (43.2 %)	6 (31.6 %)	4 (21.1 %)	0	19	1
Center 2	23 (47.9 %)	4 (17.4 %)	5 (21.7 %)	4	19	0.826
Center 3	17 (47.2 %)	2 (11.8 %)	5 (29.4 %)	2	15	0.882
Center 4	24 (66.7 %)	10 (41.7 %)	3 (12.5 %)	6	18	0.75
Center 5	25 (41.7 %)	5 (20 %)	5 (20 %)	2	23	0.92
Total	108 (48.2 %)	27 (25 %)	22 (20.4 %)	14	94	0.87

Two common VF mimics are OAw and acquired short vertebrae (SV), the ability to exclude these VF mimics is shown in Table 7. In the external test datasets, there were in total 137 cases with OAw, in this study the accuracy was 0.956 with a false positive rate of 4.4 %; there were in total 190 cases with SV, in this study the accuracy was 0.994 with a false positive rate of 0.5 %.

**Table 7:** **False positive (FP) rate for two VF mimics of osteoarthritic wedging (OAw) and acquired short vertebrae (SV).** As both OAw and SV are multiple in a subject, the data of this table is on counting case-by-case base.

Data	Total cases	OAw cases	OAw FP cases	SV cases	SV FP cases
Center 1	159	16	0	28	0
Center 2	219	44	0	56	1
Center 3	215	30	2	28	0
Center 4	169	31	2	37	0
Center 5	194	16	2	41	0
total	956	137	6 (4.4 %)	190	1 (0.5 %)

## 4 Discussion

With modern CT, some portions of the patients' spine are visualized in detail during ordinary chest and abdomen scanning, offering the opportunity for opportunistic VF detection. Academic researchers, software companies and service providers have realised the potential to identify VF as an ‘added’ service to CT scan images that have been taken for other clinical indications [26]. For example, Tomita et al. [27] described an AI enabled method to detect incidental VFs in chest, abdomen, and pelvis CT examinations. Burns et al. [28] described an AI enabled method which detects, localizes, and classifies VFs and measures BMD of thoracic and lumbar vertebral bodies on CT images. Rueckel et al. [29] described several pathology-specific AI algorithms enabled detection of relevant initially missed secondary thoracic findings in emergency whole-body CT scans, including VFs. Nicolaes et al. [30] described a study with two independent data sets of abdominal/chest CT scans of patients, with a training set of 1011 scans and a validation set of 2000 subjects. They reported the comparison of the trained Convolutional Neural Network (CNN) model with the reference readings in identifying subjects with one or more moderate or severe VF with an AUROC (area under the receiver operating characteristic curve) of 0.88, accuracy of 0.92, sensitivity of 0.81, and specificity of 0.95. Nicolaes et al. [31] additionally described a validation study with external cohorts of 4810 subjects with images acquired on 16 different CT scanners. The CNN's performance in identifying scans with ≥1 moderate or severe fractures achieved an AUROC of 0.94, an accuracy of 93 %, a sensitivity of 94 % and a specificity of 93 %. Hu et al. [32] studied using deep learning to predict subsequent VFF following an initial VFF. They retrospectively analyzed CT images from 103 patients who experienced initial VFF and subsequent VFF. CT images from 70 age-matched patients without VF were used as the negative control. For the prediction of subsequent fracture, their model attained 0.839 accuracy and AUROC 0.883 on the testing dataset.

Software companies and service providers such as Mindways (Austin, Texas, USA), ON Diagnostics (Berkeley, California, USA), Optasia Medical (Cheadle Hulme, Cheshire, UK), and Zebra Medical (Shefayim, Israel), etc. offer automated VF detection with CT image, using various technical approaches. Mindways' ‘Slicepick’ module displays anterior–posterior and lateral spine images and contains tools for identifying and confirming VF by 6-point morphometry [33]. The ‘VirtuOst’ software of O.N. Diagnostics provides a 6-point morphometry VF assessment [34]. The Optasia Medical ‘ASPIRE’ service reports VF visualized incidentally on CT. CT images are reformatted by ‘ASPIRE’ software to provide sagittal views. A 6-point placement analysis to measure the posterior, middle and anterior height of the vertebra was used to diagnose a fracture. Fracture severity was based on the percentage of height loss; grade 1 (15–25 %), grade 2 (26–40 %), and grade 3 (>40 %) [[35], (a)]. A number of articles reported the performance of Zebra Medical in analyzing chest and abdominal CT scans to automatically identify VF [[35], (a), [36], [37], [38]]. In a single-site clinical implementation study involving thoracic CT scans from 1696 patients with a VF prevalence of 24 %, Kolanu et al. [[35], (a)] reported Zebra Medical model achieved a sensitivity of 54 %, specificity of 92 % and accuracy of 83 %. In a US-based study using chest and abdominal CT scans from 1000 patients, Krishnaraj et al. [36] reported sensitivity, specificity and accuracy were 84 %, 73 % and 82 % respectively. In a study conducted in Brazil, Pereira et al. [37] included a consecutive sample of 899 chest and abdominal CT scans of patients 51–99 years of age. Scans were retrospectively evaluated by the software and by two specialists in musculoskeletal imaging for the presence of VFs with vertebral body height loss >25 %. The software showed a diagnostic accuracy of 89.6 % for moderate-to-severe VFs, with a sensitivity of 73.8 %, a specificity of 92.7 %.

Chest and abdominal CT images are initially axially presented which do not show VF well particularly for milder cases. Using automatically reconstructed sagittal images, the goal of this software is to alert a radiologist or a physician to the existence of VF and to label each and every VF visible in the scanned image data. The final diagnosis should be made by a radiologist or a physician. Compared with literature reports, our VF detection model has the following additional features: (I) our software allows the detection of VF with <20 % height loss, (II) acquired Schmorl nodes is trained to be a sign of VF, (II) endplatitis short vertebrae is not recognized as VF. We argue that 6-point morphometry may be insufficient to detect VF with <20 % vertebral height loss and endplate fracture with minimal vertebral height loss. Recent works clarified there are two types of Schmorl's nodes, i.e., congenital Schmorl nodes and acquired Schmorl nodes. Congenital Schmorl's nodes have complete cartilage endplate and bony endplate coverage, while acquired Schmorl nodes are with endplate fracture [22,39]. For subjects aged around 74 years, the prevalence of acquired SV is around 10 % for men and women, and most of the acquired SV are likely due to endplatitis [17,21]. At the age of 74 years, vertebral osteoarthritic wedging has a prevalence of around 4–6 % among Caucasian women and Chinese men [40,41]. Due to their high prevalence, endplatitis SV and osteoarthritic wedging should be differentiated from VFF, though it is not uncommon that sometimes VFF, endplatitis SV, or osteoarthritic wedging can co-exist in a vertebra. In many published articles, the reference standard of VFF is Genant SQ grading [4]. However, it is important to emphasize that it is difficult to apply Genant's SQ criteria. For example, Diacinti et al. [42] reported a study that, among 562 VFFs identified by radiologist readers in peripheral hospitals, 102 were classified as normal vertebrae by the experienced radiologist readers in a central hospital; while 205 VFFs were incorrectly evaluated by local readings as false negatives. Most Genant SQ readers use a height loss of ≥20 % as a threshold to diagnose VFF. The estimation of percentage height loss by human eye is an important source of error. In fact, among older populations, those VFs with <20 % vertebral height loss, even those with only 10 % vertebral height loss, had been commonly classified as mild or grade-1 osteoporotic VF [43], as shown in the illustrations of the article of Genant et al. [4]. With Optasia Medical ASPIRE software, grade 1 VF is with 15–25 % vertebral height loss [[35], (a)]. For older women, subjects with VFF of less than 20 % vertebral height loss have an incident VF risk higher than the subjects without any baseline VFF [17]. In a study in men, it is shown that subjects with two or more VF of <20 % vertebral height loss (i.e., minimal grade) were associated with lower BMD than subjects with one VF of 20–25 % vertebral height loss (i.e., mild grade) [44]. Despite the fact that we require our model to detect minimal grade VF, our model demonstrated an overall detection sensitivity of 0.906 and an overall detection specificity of 0.986, with an overall accuracy of 0.967. For cases with ≥ moderate grades OVF, 95.54 % were detected. As noted, most false negative cases and false positive cases were minimal or mild, or associated with artifacts, or close to the border of reconstructed images (Fig. 7, Fig. 8, Fig. 9). The results of our Ofeye 3.0 compare favorably with other published automated VFF detection models (details as shown in Table-8).

**Table 8:** Comparison with other published automated VF detection models.

	Sensitivity	Specificity	Accuracy
Kolanu et al.	54 %	92 %	83 %
Krishnaraj et al.	84 %	73 %	83 %
Pereira et al.	73 %	92 %	89 %
Our method	90 %	98 %	96 %

There are still limitations to this study. Ofeye 3.0 does not grade the severity of the VF, instead it offers a ‘yes/no’ selection and provides a VF probability estimation. Overall, a smaller vertebral height loss is associated with a smaller value of probability and vice versa. In the coming study, we will add the function of VF grading. The goal is to use OLVFss (osteoporotic-like VF sum score) to evaluate the overall severity of VFF in a study subject. For each vertebra, according to the extended semi-quantitative (eSQ) scheme [45], a score of 0, -0.5, −1, −1.5, −2, −2.5, and −3 is assigned for no OLVF or OLVF of <20 %, ≥20 %–25 %, ≥25 %–33 %, ≥33 %–40 %, ≥40 %–67 %, and ≥67 % vertebral height loss, respectively [16]. An OLVFss is calculated by summing up the scores of all vertebrae visible on a CT scan. While milder degree of fracture shaped vertebral deformity is not uncommon among populations with normal bone strength [17], for women OLVFss ≤ −1.5 statistically meets the criteria for diagnosing osteoporosis, for men OLVFss ≤ −2.5 statistically meets the criteria for diagnosing osteoporosis [16,17]. Note that men and women have very different fragility profiles. Recently, a new low BMD category of ‘osteofrailia’ for men with femoral neck T-score of ≤ -2.0 has been proposed [46]. The OLVFss threshold for diagnosing osteofrailia in men will need additional validation in future studies. Finally, our current scheme is meant to support physicians to make a clinical decision. Once a possible VF is identified, then a radiologist or a physician will need to make an adjudgment, whether this VF is a true VF and not due to possible artifact, this radiologist or a physician also needs to make the decision that this VF is ‘severe’ enough to be relevant.

In conclusion, we developed a software program, Ofeye 3.0, for automated all-inclusive VF detection on chest and/or abdominal CT image data and conducted a multi-center external validation study. Ofeye 3.0 has a very low false positivity rate (1.37 %), and also for moderate and severe VFs a low false negativity rate (4.46 %). Ofeye 3.0 also has a low false positive rate for OAw and acquired short vertebrae. Despite the challenging requirements for the software to detect all-inclusive VF, our results compare favorably with other published automated VFF detection models. As the prior Ofeye 2.0 program was trained with traumatic VF with minimal or mild deformity and current Ofeye 3.0 program emphasizes specificity, upon further improvement with emphasis on sensitivity and additional validation, our VF CT model may be also suitable for automated traumatic VF detection. Moreover, the automated CSR method developed for the current study shows advantages in demonstrating structural changes of the endplate and adjacent structures.

## Ethical approval

The retrospective testing usage of the CT image data was approved by each institutional ethical committee of the respective hospitals.

## Consent to participate

This study retrospectively used patients CT image data. Informed consent was waived for individual patient.

## Availability of data and materials

Raw data can be obtained by external researchers for analysis by contacting the corresponding author of this article.

## Author contributions

Ben-Heng Xiao, Methodology, Writing - Review & Editing, Zhen-Hua Gao, Study materials, Writing - Review & Editing, Xiao-Ming Leng, Study materials, Writing - Review & Editing, Er-Zhu Du, Study materials, Writing - Review & Editing, Jian-Bing Ma, Study materials, Writing - Review & Editing, Fu-Shan Liu, Study materials, Writing - Review & Editing, Jing-Shan Gong, Study materials, Writing - Review & Editing, Zhi-Guo Ju, Study materials, Writing - Review & Editing, Ming-Yuan Yuan, Study materials, Writing - Review & Editing, Hui-Ming Zhu, Study materials, Writing - Review & Editing, Michael S. Y. Zhu, Methodology, Writing - Original Draft, Writing - Review & Editing, Timothy YC Kwok, Writing - Original Draft, Writing - Review & Editing, Yì Xiáng J. Wáng, Conceptualization, Methodology, Writing - Original Draft, Writing - Review & Editing.

## Funding

This study was partially funded by a 10.13039/501100017649Hong Kong government ITSP fund (No. ITS/334/18). Any opinion expressed in the paper does not necessarily reflect the view of the funder.

## Declaration of competing interests

YXJW is the founder of Yingran Medicals Ltd, which develops medical image-based diagnostics software, including Ofeye 1.0, Ofeye 2.0, Ofeye 3.0. BHX and MSYZ contributed to the development of Ofeye 1.0, Ofeye 3.0. The other authors have no conflicts of interest to declare.
