# Supplementary material for: Development and multi-center validation of a software program, Ofeye 3.0, for automated all-inclusive vertebral fracture detection with chest/abdominal CT images
Source: J Orthop Translat. 2025 Sep 13;55:192–203. doi: 10.1016/j.jot.2025.08.011 (PMC12799497; doi:10.1016/j.jot.2025.08.011)
Supplement: Multimedia component 1 [file mmc1.docx]

**Supplementary material**

CSR method step by step,

Step1: i from 1 to sum number of axial images, read each DICOM file and get the axial image matrix Ai{l*w}, l represents axial image length, w represents axial image width, i represents the instance number of the CT series, and adjust the window width(300) and window level (1500) of the Ai{l*w}

Step 2: following the instance number of axial image from 1 to max instance number, reshape all the axial images’ matrix to a 3-dimensional matrix B{l*w*h}, h represents the number of total axial images.

Step 3: j from 1 to w, computing sum of all matrix Cj{l*h} elements of every sagittal image, and get the matrix D{1*w}

Step 4: find the max value index number (mvin) of the matrix D{1*w}, it’s the central sagittal image index of the matrix B{l*w*h}

Step 5: k from mvin-20 to mvin+20, get Ek{l*h}, all the 40 sagittal images of spine central part, defined central slab of sagittal image as matrix E{l*40*h}

Step 6: e from 0 to 40, sum of matrix Ee{l*h}, get the final central slab reconstruction of sagittal image matrix F{l*h}

Step 7: Based on thickness (s) and pixel spacing (ps), use bilinear interpolation method to recover the image real length and width (L equals to h*s, W equals to l*ps), get the CSR image matrix G{L*W}.

Step 8: find the max and minimal element value of G{L*W} to standardize elements to 0-1, defined as Gs{L*W}

Step 9: H{L*W} equal to Gs{L*W}*255, and convert all elements of H{L*W} as int8

Step 10: save CSR image matrix H{L*W} as PNG file.

| Data source | Manufacture (percentage) | Scan types | X-Ray Tube Voltage (Kv) / tube current (mA) | Thickness (mm) | Pitch | Pixel spacing  (mm) |
| --- | --- | --- | --- | --- | --- | --- |
| Center 1 | SIEMENS (6.28%) | Chest | 120/ (186,183,191, 1566, 1565),  80/431,  100/ (388,591) | 1.0, | 1.2, 3.0 | 0.533-0.697 |
|  |  | Abdomen | 100/188,  140/557 | 1.0 | 0.8, 1.0 | 0.675-0.742 |
|  |  |  |  |  |  |  |
|  | TOSHIBA (1.25%) | Unknown | 120/ (152, 196) | 1.0 | 0.813 | 0.625,0.694 |
|  | UIH (92.45%) | Chest | 120/ (332, 91) | 0.625, 1.2 | 1.112 | 0.509-0.796 |
|  |  | Abdomen | 120/ (153, 165, 536, 220, 278, 226, 103, 304, 335, 353, 461, 260, 262, 242) | 1.0,5.0 | 0.987 | 0.607-0.818 |
| Center 2 | SIEMENS (2.73%) | Chest | 120/ (295, 216, 152, 298, 212, 299) | 1.0 | 0.6 | 0.734, 0.730 0.570 |
|  |  | Abdomen | 120/62 | 1.0 | 0.8 | 0.576 |
|  | GE (97.26%) | Chest | 100/ (220, 170, 221, 225, 276, 207, 214, 306, 284, 151, 215, 188, 280, 217, 159, 144, 321, 141, 287, 229, 265, 193, 176, 185, 130, 194, 165, 184, 168, 248, 181, 166, 286, 385, 129, 274, 281, 177, 278, 244, 262, 263, 261, 157, 239, 211, 142, 323, 172, 417, 164, 269, 358, 148, 210, 231, 367, 163, 243, 187, 212, 453, 138, 154, 197, 241, 240, 199, 175, 223, 296, 246, 135, 182, 179, 162, 146, 191, 257, 137, 237, 302, 192, 203, 200, 173, 180, 161, 160, 226, 227, 349)  120/ (136, 250, 248, 98, 173, 93, 294, 216, 91, 344, 100, 88, 106, 211, 89, 80, 346, 138, 183, 144, 101, 209, 325, 255, 201, 301, 99, 117, 86, 131, 113, 92) | 1.25, 0.625 | 0.992 | 0.574-0.853 |
|  |  | Abdomen | 120/405 | 1.25 | 0.992 | 0.666-0.957 |
| Center 3 | SIEMENS (13.95%) | Abdomen | 70/480  90/ (276, 278, 273, 286, 446, 447, 453, 269, 285, 284,)  100/(264, 289,288, 209, 220, 223, 224) | 1.0, | 1.0, 0.6, 1.2 | 0.531-0.826 |
|  |  | Chest | 90/ (257, 530, 547,529, 582, 251, 610, 330, 320, 190,318,)  110/ (340, 331, 319, 408, 529, 318, 407, 1938,1936)  120/ (332, 372, 1306, 413, 255, 371, 1304, 186, 254, 414, 389, 350, 422, 1997, 1723, 1720, 265, 1270, 435, 1996) | 1.0 | 0.6, 1.0, 1.2, 1.55, 1.6, 1.9 | 0.578-0.933 |
|  |  | Heart | 100/754  120/480 | 0.75, 1.0, 3.0 | 0.27 | 0.324 |
|  | GE (6.04%) | Abdomen | 120/351 | 1.0 | 0.992 | 0.707 |
|  |  | Chest | 70/ (319, 594, 521, 459)  80/(397, 347, 292, 551) | 1.0 | 0.992 | 0.583-0.728 |
|  |  | Unknown | 120/ (201, 271)  140/515 | 2.79, 3.75, 1.25, 5.00, 0.625 | 0.984, 1.374 | 0.488-1.953 |
|  | TOSHIBA (53.95%) | Chest | 100/ (173, 169,)  120/ (200, 50, 191, 115, 497, 300, 80, 105, 100, 309, 178, 173, 117, 179, 250, 109, 146, 103, 149, 253, 402, 467, 145, 380, 138, 132, 400, 209, 206, 112, 104, 176, 159, 127, 197, 114, 99, 110,177, 141) | 0.5, 1.0 | 0.813, 1.388, 1.484 | 0.404-0.873 |
|  |  | Abdomen | 120/ (141, 101, 200, 173, 250, 120, 136, 300, 80, 107, 222, 184, 104, 113, 100, 178, 130, 176, 175, 194, 197, 211, 154, 150, 127, 469, 225, 135, 179, 160, 500, 172, 148, 170, 245, 143, 217, 232) | 0.5, 1.0, 3.0 | 0.641, 0.638, 0.813, 0.828 | 0.515-0.805 |
|  |  | Unknown | 120/ (135, 250, 193, 115, 200, 104, 100) | 1.0 | 0.638, 0.813 | 0.552-0.743 |
|  | PHILIPS (24.65%) | Chest | 120/ (401, 397, 360, 338, 417, 435, 347, 423, 411, 404, 254, 408, 520, 290, 284, 427, 297, 332, 398, 348, 313, 244, 320, 450, 270, 315, 368, 324, 269, 299, 333, 287, 294, 442, 392, 365, 372, 316, 319, 321, 307, 301, 317) | 1.0 | 0.843-1.224 | 0.632-0.826 |
|  |  | Abdomen | 120/ (337, 340, 336, 254, 492, 247, 494, 493, 166, 332, 442, 445, 321, 242, 163, 213, 164) | 1.0 | 1.234, 0.891 | 0.652-0.853 |
|  | CANON (1.39%) | Chest | 120/ (150, 132) | 1.0 | 1.388 | 0.705,0.782 |
|  |  | Abdomen | 120/110 | 1.0 | 0.813 | 0.633 |
|  |  | Unknown | 120/135 | 1.0 | 0.813 | 0.728 |
| Center 4 | SIEMENS (100%) | Chest | 100/ (110, 109, 225, 179)  120/(141, 156, 140, 122, 155, 131, 112, 144, 134, 147, 120, 79, 137, 117, 133, 126,78, 143, 177, 242, 135, 310, 283, 262, 176, 148, 139, 150, 180, 219, 145, 153, 90, 160, 165, 168,104, 119, 124, 151, 381, 130, 171, 129, 161, 181, 127, 149, 95, 121, 136, 309, 346, 291, 201, 208, 125, 271, 111, 272, 189, 363, 229, 163, 190, 138, 210, 123, 243, 172, 159, 175, 617, 158, 110, 162, 615, 222, 709, 169, 114, 142, 77, 152, 423, 132, 166, 185, 179, 254, 196, 223, 528)  130/ (124, 93, 139,148, 144, 105, 116, 145, 104, 96) | 1.0, 1.5, 2.0, 5.0 | 1.2, 0.6, 0.8, 1.1, 0.9, 1.5, 0.7, 0.85, 0.95, 3.0, 1.05, 1.0, 1.3 | 0.396-0.871 |
|  |  | Abdomen | 120/ (192, 145, 161, 199, 200, 295, 198, 197,205) | 0.6, 2.0 | 0.6 | 0.583-0.890 |
|  |  | Spine | 130/ (105, 100, 88, 140) | 0.6, 5.0 | 0.8 | 0.349-0.433 |
|  |  | Heart | 100/ (746, 831, 1099)  120/ (221, 182, 269) | 0.6, 3.0 | 0.23, 0.26, 0.24, 0.32, 0.33 | 0.302-0.355 |
|  |  |  |  |  |  |  |
| Center 5 | SIEMENS (71.13%) | Chest | 120/ (664, 640, 622, 540) | 1.0 | 0.8, 0.75, 0.55 | 0.572-0.966 |
|  |  | Abdomen | 120/ (640, 600, 636, 604, 520) | 1.0 | 0.8, 0.75, 1.0, 0.95, 0.65 | 0.683-0.962 |
|  |  | Pelvis | 140/520  120/640 | 1.0 | 0.65, 0.8 | 0.736-0.841 |
|  | GE (28.86%) | Unknown | 120/ (500, 450, 350, 400, 525, 450) | 1.25 | 1.375, 0.984 | 0.654-0.976 |

Details of scanning procotool (*UIH, United Imaging Healthcare; SIEMENS, Siemens Healthineers; TOSHIBA, Toshiba Medical Systems Corporation; GE, GE MEDICAL SYSTEMS; CANON, Canon Medical Systems)
